# Retraction Note: Comparative study between oxytocin and combination of tranexamic acid and ethamsylate in reducing intra-operative bleeding during emergency and elective cesarean section after 38 weeks of normal pregnancy

**DOI:** 10.1186/s12884-025-08322-4

**Published:** 2025-10-14

**Authors:** Almandouh H. Bosilah, Elsayed Eldesouky, Moatazza Mahdy Alghazaly, Elsayed Farag, Eslam Elsayed Kamal Sultan, Hosam Alazazy, Attia Mohamed, Soliman Mohamed Said Ali, Ahmed Gamal Abo Elsror, Mohamed Mahmoud, Abd Elhalim Mohamed Abd Elhalim, Mohamed Abdelmonem Kamel, Mohamed Abd-ElGawad, Fatma Mohamed Sayed, Mohamed Sobhy Bakry

**Affiliations:** 1 Department of Obstetrics and Gynecology, Faculty of Medicine, Damietta University, Damietta, Egypt; 2 Department of Obstetrics and Gynecology, Faculty of Medicine, Alazhar University, Cairo, Egypt; 3 Department of Obstetrics and Gynecology, Faculty of Medicine, Alazhar University for Girls, Cairo, Egypt; 4 Department of Obstetrics and Gynecology, Faculty of Medicine, Alazhar University, Domiata, Egypt; 5 International Islamic Center for Population Research, Alazhar University, Cairo, Egypt; 6 Department of Obstetrics and Gynecology, Faculty of Medicine, Alazhar University, Assuit, Egypt; 7 Faculty of Medicine, Fayoum University, Fayoum, Egypt; 8 Department of Obstetrics and Gynecology, Faculty of Medicine, Fayoum University, Fayoum, Egypt

**Retraction Note: BMC Pregnancy and Childbirth 23, 433 (2023)**

**https://doi.org/10.1186/s12884-023-05728-w**

RETRACTION NOTE

The Editor has retracted this article after concerns were raised about the recruitment and methodology reported in the study. The authors sent their original data, and after post-publication assessment the data was found to contain irregularities. For instance, the same amount of blood loss appears to happen with an unusual frequency, and out of 100 cases reported, there are 24 pairs of patients where the data appears to be unusually similar. Authors Mohamed Abd-ElGawad, Fatma Mohamed Sayed Mohamed Abdelmonem Kamel confirmed that there were issues with the data and asked the Editor to retract the paper. Mohamed Abd-ElGawad agrees to this retraction. Almandouh H. Bosilah, Elsayed Eldesouky, Moatazza Mahdy Alghazaly, Elsayed Farag, Eslam Elsayed Kamal Sultan, Hosam Alazazy, Attia Mohamed, Soliman Mohamed Said Ali, Ahmed Gamal Abo Elsror, Mohamed Mahmoud, Abd Elhalim Mohamed Abd Elhalim, Mohamed Abdelmonem Kamel, Fatma Mohamed Sayed and Mohamed Sobhy Bakry did not reply whether they agree to this retraction.

